# Supplementary material for: Hydroethanolic extract of Schinus terebinthifolia as a promising source of anti-influenza agents: Phytochemical profiling, cheminformatics, molecular docking and dynamics simulations
Source: PLoS One. 2025 Jun 2;20(6):e0324990. doi: 10.1371/journal.pone.0324990 (PMC12129213; doi:10.1371/journal.pone.0324990)
Supplement: Attachment — Submitted filename: Response to Reviewers - PONE-D-24-35830.docx [file pone.0324990.s002.docx]

### Faculty of Pharmaceutical Sciences, Khon Kaen University

## Division of Pharmacognosy and Toxicology, Khon Kaen 40002, Thailand

**April 22, 2025**

**Dear Editor and Reviewers,**

Firstly, we would like to inform you that we have decided to revise the title from “Hydroethanolic extract of *Schinus terebinthifolia* as a promising source of anti-influenza agents: Phytochemical profiling, cheminformatics, and *in silico* docking assessments” to “Hydroethanolic extract of *Schinus terebinthifolia* as a promising source of anti-influenza agents: Phytochemical profiling, cheminformatics, molecular docking and dynamics simulations”, to better reflect the inclusion of molecular dynamics simulation studies.

Secondly, thank you very much to editor and reviewers for the revision of our manuscript title “**Hydroethanolic extract of *Schinus terebinthifolia* as a promising source of anti-influenza agents: Phytochemical profiling, cheminformatics, molecular docking and dynamics simulations**”. We would like to explain point-by-point the details of the revisions in the manuscript and our responses to the reviewers' comments. The revisions that have been made to the manuscript were also included as followed:

**Review Comments to the Author**

**Reviewer #1**

The manuscript titled "Hydroethanolic extract of *Schinus terebinthifolia* as a promising source of anti-influenza agents" presents interesting findings regarding the antiviral properties of the plant extract. However, several areas could be improved for clarity, depth, and overall impact. Here are some comments:

1. The introduction should provide a clearer rationale for the study. While it mentions the biological activities of *Schinus terebinthifolia*, it could benefit from a more detailed discussion on the significance of exploring its anti-influenza properties specifically. This would help contextualize the research better for readers unfamiliar with the subject.

**Response:** We have added and revised the reason for exploring its anti-influenza properties in the introduction as follows:

“Humans are vulnerable to a variety of viral illnesses, one of which is H1N1, which poses a significant threat to public health. The high morbidity and mortality rates associated with this disease make it a pressing issue that requires attention. Viral diseases are not limited to a specific geographic region and can cause severe health problems in both children and the elderly. Although vaccines are available, influenza still causes an estimated 250,000–500,000 deaths annually in industrialized countries, with 6.1 deaths per 100,000 population reported in Thailand, partly due to the virus's ability to develop drug resistance through mutations [1,2]. Hence, there is a need for new medicines, and plants offer a promising source due to their rich secondary metabolites with antioxidant and broad therapeutic potential against both infectious and non-infectious diseases.” Line 46-54

**References**

[1] B. S. Cooper, S. Kotirum, W. Kulpeng, N. Praditsitthikorn, M. Chittaganpitch, D. Limmathurotsakul, N. P. J. Day, R. Coker, Y. Teerawattananon, A. Meeyai, Mortality Attributable to Seasonal Influenza A and B Infections in Thailand, 2005–2009: A Longitudinal Study, American Journal of Epidemiology, 181(11) (2015) 898-907, <https://doi.org/10.1093/aje/kwu360>.
[2] K. Prasert, P. Praphasiri, S. Nakphook, D. Ditsungnoen, P. Sapchookul, K. Sornwong, S. Naosri, P. Akkapaiboon Okada, P. Suntarattiwong, T. Chotpitayasunondh, M. P. Montgomery, W. W. Davis, C. Pittayawonganon, Influenza virus circulation and vaccine effectiveness during June 2021-May 2023 in Thailand, Vaccine X, 19 (2024) 100517, <https://doi.org/10.1016/j.jvacx.2024.100517>.

2. The methods section lacks sufficient detail regarding the extraction process and the specific conditions used (e.g., temperature, duration). Providing this information would enhance reproducibility and allow other researchers to replicate the study more effectively.

**Response:** In the methods section (Preparation of *S. terebinthifolia* aqueous ethanol extract), We have provided the specific condition used for extraction process as follows:

*2.1 Preparation of S. terebinthifolia aqueous ethanol extract*

Fresh materials of *S. terebinthifolia*, including leaves and stalks, were harvested from an open field in Nam Phong subdistrict, Khon Kaen province, Thailand (16°37'59.9" N, 102°46'14.3" E). The collected plant materials were rinsed thoroughly with tap water multiple times and then soaked twice in deionized water for 60 minutes each to ensure all residues were removed. The plant materials were dried at 50°C for 2-3 days in an oven incubator (Model KT(E6), BINDER, Neckarsulm, Germany). Once completely dried, they were ground into a fine powder using a blender. For the hydro-ethanolic extraction, 20 g of the powdered plant material was placed in a 500 mL Erlenmeyer flask and extracted with 200 mL of 30% (v/v) hydroethanolic solution. The extraction was performed by gently shaking at 100 rpm at 25°C for 48 hours in an incubator shaker (Model NB-250VL, N-BIOTEK, Gyeonggi-do, South Korea). The supernatant was separated from the plant extract at 25°C and 8,000 rpm for 20 minutes using ST1R Plus-MD Refrigerated Centrifuge (Thermo Scientific, Massachusetts, USA). The clear supernatant was transferred to a round-bottom flask, and approximately 60 mL of ethanol was removed using a Rotavapor R-3 Laboratory Evaporator (BUCHI, Finland). The 140 mL water-based solution was freeze-dried at −110°C for 48 hours using a CoolSafe freeze dryer (Labogene, Allerød, Denmark) and stored at −80°C until analysis. Line 99-115

3. The results could be presented more clearly. The HPLC analysis results for gallic acid should include a more comprehensive discussion on the implications of the yield (1.71 mg/g DW) and how it compares to other studies. Additionally, including visual aids such as graphs or tables would help in conveying the data more effectively.

**Response:**

Table 1 presents data on the gallic acid content and total phenolic content (TPC) of various plant extracts obtained using different extraction solvents. Several studies have demonstrated that aqueous-ethanolic plant extracts can serve as promising sources of antiviral agents [1]. In our study, as well as in others summarized in Table 1, hydroethanolic and water-based solvents were used for plant extraction due to their suitable polarity and safety profile. There are variations in gallic acid and total phenolic content are influenced in each study because of several factors such as plant species, specific plant part used, type of extraction solvent, and extraction method.

**Table 1.** Gallic acid content in various plan extracts

|  | **Plant source** | **Solvent for extraction** | **Gallic acid content** | **Total phenolic content** | **References** |
| --- | --- | --- | --- | --- | --- |
| 1 | *Schinus terebinthifolia* | 30% Ethanol in water | 1.71 mg/g DW | 264.11 (GAE)/g DE | This study |
| 2 | *Caesalpinia mimosoides Lamk* | water | 8.77 mg/g DW | 551 mg (GAE)/g DE | Klamrak et al., 2023^(2)^ |
|  |  | 30% Ethanol in water | 7.12 mg/g DW | 337 mg (GAE)/g DE | Klamrak et al., 2025^(3)^ |
| 4 | *Mangifera indica* | 20% Ethanol in water | 6.1 mg/g DW | 64.30 mg (GAE)/g DE | Hayat et al., 2023^(4)^ |
| 5 | *Caesalpinia spinosa* | water | 33.4 mg/g DW | 349.7 mg (GAE)/g DE | Skowyra et al., 2013^(5)^ |
|  |  | 50% Ethanol in water | 22.3 mg/g DW | 445.3 mg (GAE)/g DE |  |
|  |  | 70% Ethanol in water | 16.9 mg/g DW | 460.2 mg (GAE)/g DE |  |
| 6 | *Toona sinensis* | water |  | 0.13 mg (GAE)/g FE^*^ | Yang et al., 2005^(6)^ |

*Fresh extract

**References**

[1] K. Chojnacka, D. Skrzypczak, G. Izydorczyk, K. Mikula, D. Szopa, A. Witek-Krowiak, Antiviral properties of polyphenols from plants, Foods, 10(10) (2021) 2277, <https://doi.org/10.3390/foods10102277>.
[2] A. Klamrak, J. Nabnueangsap, J. Narkpuk, Y. Saengkun, P. Janpan, N. Nopkuesuk, A. Chaveerach, S. Teeravechyan, S.S. Rahman, T. Dobutr, P. Sitthiwong, Unveiling the Potent Antiviral and Antioxidant Activities of an Aqueous Extract from *Caesalpinia mimosoides* Lamk: Cheminformatics and Molecular Docking Approaches, Foods, 13(1) (2023) 81, <https://doi.org/10.3390/foods13010081>.
[3] A. Klamrak, S.S. Rahman, N. Nopkuesuk, J. Nabnueangsap, J. Narkpuk, P. Janpan, Y. Saengkun, T. Soonkum, S. Sriburin, S. Teeravechyan, P. Sitthiwong, N. Jangpromma, S. Kulchat, K. Choowongkomon, R. Patramanon, A. Chaveerach, J. Daduang, S. Daduang, Integrative computational analysis of anti-influenza potential in Caesalpinia mimosoides Lamk hydroethanolic extract, Scientific reports, 15(1) (2025) 3988, <https://doi.org/10.1038/s41598-025-87585-5>.
[4] Z. Hayat, T. Riaz, K. Saleem, K. Akram, H.U. Rehman, M. Azam, Optimization of Gallic Acid-Rich Extract from Mango (Mangifera indica) Seed Kernels through Ultrasound-Assisted Extraction, Separations, 10(7) (2023) 376, <https://doi.org/10.3390/separations10070376>.
[5] M. Skowyra, V. Falguera, G. Gallego, S. Peiró, M. P. Almajano, Antioxidant properties of aqueous and ethanolic extracts of tara (Caesalpinia spinosa) pods in vitro and in model food emulsions, Journal of the Science of Food and Agriculture, 94(5) (2014) 911-8, <https://doi.org/10.1002/jsfa.6335>.
[6] H.L. Yang, W.H. Chang, Y.C. Chia, C.J. Huang, F.J. Lu, H.K. Hsu, Y.C. Hseu, Toona sinensis extracts induces apoptosis via reactive oxygen species in human premyelocytic leukemia cells, Food and Chemical Toxicology, 44(12) (2006) 1978-88, <https://doi.org/10.1016/j.fct.2006.06.027>.

4. The discussion section should delve deeper into the implications of the findings. While it mentions the potential of gallic acid and other metabolites as anti-influenza agents, it could explore the mechanisms of action in more detail. This would provide a better understanding of how these compounds interact with viral targets like neuraminidase and PB2.

**Response:** Thank you for your insightful comment. We have now provided additional details regarding the proposed mechanisms of action of gallic acid and other metabolites as potential anti-influenza agents. These explanations have been integrated into the Results and Discussion. A summary of the key points is provided below:

The previous study suggests a strong correlation between phenolic compounds found in specific plant extracts and their antiviral properties. These compounds may interact with viral protein targets—such as neuraminidase (NA), hemagglutinin (HA), and the PB2 subunit of RNA polymerase—through hydrogen bonding and hydrophobic interactions [1]. In this study, the hydroethanolic extract of *S. terebinthifolia* exhibited a total phenolic content (TPC) of 264.11 mg GAE/g dry extract (DE), with gallic acid specifically quantified at 1.71 mg/g DE. Through untargeted metabolomics analysis coupled with a series of cheminformatics tools (MetFrag, SIRIUS, CSI:FingerID, and CANOPUS), we tentatively identified and annotated 24 bioactive metabolites within the extract (Table 2 in the manuscript).

In the antiviral assay, the *S. terebinthifolia* hydroethanolic extract demonstrated an IC₅₀ of 2.21 µg/mL against influenza A/PR/8/34 virus, making it approximately 1.96 times more effective than gallic acid alone (IC₅₀ = 4.35 µg/mL). A preliminary estimation showed that the gallic acid content in the extract was around 0.27 µg/mL, suggesting that its contribution alone could not account for the observed antiviral potency. This suggests that other phytochemicals may contribute to a synergistic effect in this circumstance. Moreover, the extract exhibited inhibitory effects across multiple stages of the viral life cycle.

In the virtual screening for anti-influenza potential, computer-aided molecular docking revealed that several metabolites from the extract interacted with the catalytic site of NA. Additionally, in silico docking showed that metabolites such as myricitrin, afzelin, chlorogenic acid, and methyl- and ethyl-gallate exhibited strong binding affinities toward the PB2 subunit, comparable to reference ligands such as gallic acid, quercetin, and hyperoside (Table 4 in the manuscript). These findings align with previous studies showing that metabolites derived from the shikimate and phenylpropanoid pathways—such as gallic acid, quercetin, and glycosylated analogs—can specifically bind to the cap-binding domain of PB2, forming hydrophobic interactions with conserved residues (e.g., Phe323, His357, Phe363, and Phe404), thereby disrupting viral replication [2,3]. Therefore, NA and PB2 appear to be key targets underlying the anti-influenza activity of the hydroethanolic extract. Furthermore, molecular dynamics (MD) simulation results confirmed the stability of interactions between these viral proteins (NA and PB2) and the top three selected phytochemicals from the extract, thereby validating the docking findings (Figs. 14 and 15 in the manuscript). These combined in silico approaches suggest that the selected metabolites may serve as potential inhibitors of viral proteins such as neuraminidase and the PB2 subunit of RNA polymerase.

**References**

[1] H.L. You, C.C. Huang, C.J. Chen, C.C. Chang, P.L. Liao, S.T. Huang, Anti-pandemic influenza A
     (H1N1) virus potential of catechin and gallic acid, Journal of the Chinese Medical Association,
     81(5) (2018) 458-68, DOI: [10.1016/j.jcma.2017.11.007](https://doi.org/10.1016/j.jcma.2017.11.007).
[2] P. Mehrbod, D. Hudy, D. Shyntum, J. Markowski, M.J. Łos, S. Ghavami, Quercetin as a natural
     therapeutic candidate for the treatment of influenza virus, Biomolecules, 11(1) (2020) 10,
     <https://doi.org/10.3390/biom11010010>.
[3] E. Gansukh, Z. Kazibwe, M. Pandurangan, G. Judy, D.H. Kim, Probing the impact of quercetin-7-
     O-glucoside on influenza virus replication influence, Phytomedicine, 23(9) (2016) 958-67,
     <https://doi.org/10.1016/j.phymed.2016.06.001>.

5. The manuscript should explicitly state the limitations of the study, such as the in vitro nature of the findings and the need for *in vivo* studies to confirm efficacy.

**Response:** Thank you for your suggestion, which highlights the limitations of this manuscript. We are conducting the studies to confirm the efficacy of the secondary metabolites in the aqueous ethanolic extract of *S. terebinthifolia*, particularly focusing on their anti-H5H1 properties through in vivo experiments. Line 785-788

**6. Molecular dynamic studies shall be performed for top 03 ligands for 100ns to confirm the stability.**

**Response: Line 727-769**

A 100 ns molecular dynamics (MD) simulation was performed to evaluate the stability of interactions between the target proteins PB2 and NA and their top three ligands chosen from the S. terebinthifolia aqueous ethanolic extract, validating the docking results (Figs. 1 and 2). Regarding the PB2–ligand complexes, myricitrin (1st), afzelin (2nd), hexadecanedioic acid (3rd), and quercetin (control ligand) were found to properly fit within the cap-binding site of the PB2 subunit, with respective RMSD values of 1.30, 1.92, 1.68, and 1.55 nm, indicating sufficient stability of each protein-ligand complex throughout the simulation (Fig. 1A). The average RMSD values for the NA protein with the selected ligands—afzelin (2^nd^), hexadecanedioic acid (3rd), undecanedioic acid (4^th^), and quercetin (control ligand)—were 1.71, 1.63, 1.55, and 1.79 nm, respectively (Fig. 2A). Despite slight fluctuations observed during the simulation, the results indicated overall stability, with the complexes maintaining consistently low RMSD values under the given conditions. Protein residue flexibility was also analyzed through root mean square fluctuation (RMSF), which measures the positional changes of each residue as it interacts with its ligand over the simulation period. Obviously, the four ligands—myricitrin, afzelin, hexadecanedioic acid, and quercetin—showed promising RMSF values of 0.12, 0.14, 0.13, and 0.13 nm, respectively, while remaining bound to the cap-binding residues (Phe323, His357, Phe363, and Phe404), with low fluctuations observed throughout the 100 ns simulation (Fig. 1B). Similarly, the average RMSF values for the NA protein with afzelin, hexadecanedioic acid, undecanedioic acid, and quercetin (control) were 0.11, 0.10, 0.11, and 0.10 nm, respectively, suggesting only slight fluctuations in the individual amino acid residues of this viral surface protein (Fig. 2B). These results indicate that all protein–ligand complexes involving the two viral target proteins are structurally stable, with minimal fluctuations in the core binding regions of conserved amino acids, while remaining consistently positioned within the drug recognition sites. Equally important, both NA and PB2 exhibited lower radius of gyration and solvent-accessible surface area (SASA) values (Figs. 1D and 2D), clearly indicating that the protein–ligand complexes maintained a stable conformation with no significant alterations under solvent-exposed conditions. PB2–ligand complexes displayed Rg values of 1.60, 1.61, 1.60, and 1.60 nm for myricitrin, afzelin, hexadecanedioic acid, and quercetin in that order; NA–ligand complexes exhibited values of 1.99, 1.97, 1.98, and 1.97 nm for afzelin, hexadecanedioic acid, undecanedioic acid, and quercetin accordingly (Figs. 1C and 2D). Overall, our selected phytochemicals from the hydroethanolic extract are structurally stable, as indicated by negligible fluctuations in various parameters measured at the druggable targets of the two influenza proteins.

**Fig. 1** MD simulation analysis of PB2 subunit with selected metabolites from hydroethanolic *S. terebinthifolia* extracts (Quercetin (control), Myricitrin, Afzelin, Hexadecanedioic acid). A) RMSD. B) RMSF. C) Radius of Gyration. D) Solvent accessible surface area.

**Fig. 2** MD simulation analysis of NA protein with selected metabolites from hydroethanolic *S. terebinthifolia* extracts (Quercetin (control), Afzelin, Hexadecanedioic acid, Undecanedioic acid). A) RMSD. B) RMSF. C) Radius of Gyration. D) Solvent accessible surface area.

7. Authors are suggested to add following references;
<https://doi.org/10.1016/j.heliyon.2023.e15952>

- Phytochemical profile, antioxidant, cytotoxic and anti-inflammatory activities of stem bark extract and fractions of Ailanthus excelsa Roxb.: In vitro, in vivo and in silico approaches
<https://doi.org/10.1080/14786419.2021.2013839>

- Evaluation of phytochemicals, enzyme inhibitory, antibacterial and antioxidant effects of Psydrax dicoccos Gaertn
<https://doi.org/10.1016/j.sajb.2023.01.005>

- A comparative analysis of leaf essential oil profile, in vitro biological properties and in silico studies of four Indian Guava (Psidium guajava L.) cultivars, a promising source of functional food
<https://doi.org/10.1080/10496475.2019.1689542>

- Effect of Explant Source, Culture Media, and Growth Regulators on Callogenesis and Expression of Secondary Metabolites of Curcuma Longa

**Response:** Thank you for your valuable suggestions. While the recommended references are of high scientific merit, we believe they are not directly relevant to the scope of our manuscript, particularly in terms of the plant species, specific metabolites investigated and anti-influenza activity. Therefore, we have decided not to include them in the current version.

**Reviewer #2**

1. Please add more details in introduction part to show research impact.
1.1 Provide justification for selecting Ethanol improve images quality (Special HPLC results)

**Response:** We have made additions and revisions according to the reviewer’s suggestions. Line 88-90

Several studies have shown that plant extracts, including aqueous and ethanolic types, can be promising sources of antiviral agents. This study investigated the antiviral potential of a hydroethanolic extract of *S. terebinthifolia*, inspired by prior findings that a 30% (v/v) aqueous-ethanol extract of *C. mimosoides* was more effective than aqueous extracts [1,2]. Moreover, the hydroethanolic solvent offers a balanced polarity and is considered less toxic and safer for human use than many other organic solvents. Additionally, gallic acid was used as a representative marker in this study due to its known anti-influenza activity. Therefore, in the HPLC analysis, gallic acid was dissolved in the same hydroethanolic solvent.

**References**

[1] A. Klamrak, J. Nabnueangsap, J. Narkpuk, Y. Saengkun, P. Janpan, N. Nopkuesuk, A. Chaveerach, S. Teeravechyan, S.S. Rahman, T. Dobutr, P. Sitthiwong, Unveiling the Potent Antiviral and Antioxidant Activities of an Aqueous Extract from *Caesalpinia mimosoides* Lamk: Cheminformatics and Molecular Docking Approaches, Foods, 13(1) (2023) 81, <https://doi.org/10.3390/foods13010081>.
[2] A. Klamrak, J. Nabnueangsap, J. Narkpuk, Y. Saengkun, P. Janpan, N. Nopkuesuk, A. Chaveerach,
     S. Teeravechyan, S.S. Rahman, T. Dobutr, P. Sitthiwong, Unveiling the Potent Antiviral and
     Antioxidant Activities of an Aqueous Extract from *Caesalpinia mimosoides* Lamk:
     Cheminformatics and Molecular Docking Approaches, Foods, 13(1) (2023)
     81,<https://doi.org/10.3390/foods13010081>.

**1.2 Please add the catalog number for standard reference for HPLC chemicals used in the recent study.**

**Response:** We have added the information of the standard reference (gallic acid) for HPLC as follows: Gallic acid (3,4,5-Trihydroxybenzoic acid), Product No. G7384, Sigma-Aldrich, Missouri, USA.

Line 119-120

**1.3 Improve conclusion.**

**Response:** We have made revisions according to the reviewer’s suggestions. Line 770-788

**Conclusion**

As a new finding, our study indicates that the hydroethanolic extract (30% v/v ethanol solution) prepared from the aerial parts of S. terebinthifolia is a promising source of antioxidant and anti-influenza agents. Additionally, the newly established plant extract was found to be free of hemolytic activity, suggesting that it may be a safe antiviral agent. Gallic acid could be representatively detected from this phytochemical extract, according to RP-HPLC analysis. Notably, the use of high-resolution LC-ESI(±)-QTOF-MS/MS, with the aid of cheminformatics software, revealed that it contains not only gallic acid but also other relevant metabolites in the shikimate and phenylpropanoid pathways, such as methyl gallate, ethyl gallate, quinic acid, shikimic acid, chlorogenic acid, myricitrin, and afzelin. Additionally, dicarboxylic acids and a putative germacrone were also identified. The selected phytochemicals from the hydroethanolic extract demonstrated strong drug-likeness, aligning with Lipinski’s and Ghose’s rules, thereby indicating their therapeutic potential. *In silico* docking studies identified neuraminidase (NA) and the cap-binding domain of influenza RNA polymerase (PB2) as promising inhibitory targets, supporting the extract's antiviral activity. Molecular docking and MD simulations further confirmed the binding stability of representative natural product classes within the extract. Given the broad-spectrum antiviral properties of phenolic compounds, particularly against various influenza pathways, ongoing investigations are assessing the extract's efficacy against H5N1—a strain currently posing a global health threat.

We thank for your time in reviewing our manuscript and look forward to meeting your expectations.

Sincerely,

Prof. Dr. Sakda Daduang

Division of Pharmacognosy and Toxicology, Faculty of Pharmaceutical Sciences, Khon Kaen University, Khon Kaen 40002, Thailand
